# Hydroxychloroquine in patients with inflammatory and erosive osteoarthritis of the hands (OA TREAT): study protocol for a randomized controlled trial

**DOI:** 10.1186/1745-6215-15-412

**Published:** 2014-10-27

**Authors:** Jacqueline Detert, Pascal Klaus, Joachim Listing, Vera Höhne-Zimmer, Tanja Braun, Siegfried Wassenberg, Rolf Rau, Frank Buttgereit, Gerd R Burmester

**Affiliations:** Department of Rheumatology and Clinical Immunology, Charité-Universitätsmedizin Berlin, Charitéplatz 1, 10117 Berlin, Germany; German Rheumatism Research Center, Berlin, Charitéplatz 1, 10117 Berlin, Germany; Rheumatologic Department, Evangelisches Fachkrankenhaus, Rosenstraße 2, 40882 Ratingen, Germany; Specialist of Radiology in Rheumatology, Irisweg 5, 40489 Düsseldorf, Germany

**Keywords:** Erosive hand osteoarthritis, Hydroxychloroquine, Double-blind, Hand, Placebo-controlled, Randomized

## Abstract

**Background:**

Osteoarthritis (OA) is a heterogeneous group of conditions with disturbed integrity of articular cartilage and changes in the underlying bone. The pathogenesis of OA is multifactorial and not just a disease of older people. Hydroxychloroquine (HCQ) is a disease-modifying anti-rheumatic drug (DMARD) typically used for the treatment of various rheumatic and dermatologic diseases. Three studies of HCQ in OA, including one abstract and one letter, are available and use a wide variety of outcome measures in small patient populations. Despite initial evidence for good efficacy of HCQ, there has been no randomized, double-blind, and placebo-controlled trial in a larger patient group. In the European League Against Rheumatism (EULAR), evidence-based recommendations for the management of hand OA, HCQ was not included as a therapeutic option because of the current lack of randomized clinical trials.

**Methods/Design:**

OA TREAT is an investigator-initiated, multicenter, randomized, double-blind, placebo-controlled trial. A total of 510 subjects with inflammatory and erosive hand OA, according to the classification criteria of the American College of Rheumatology (ACR), with recent X-ray will be recruited across outpatient sites, hospitals and universities in Germany. Patients are randomized 1:1 to active treatment (HCQ 200 to 400 mg per day) or placebo for 52 weeks. Both groups receive standard therapy (non-steroidal anti-inflammatory drugs [NSAID], coxibs) for OA treatment, taken steadily two weeks before enrollment and continued further afterwards. If disease activity increases, the dose of NSAID/coxibs can be increased according to the drug recommendation. The co-primary clinical endpoints are the changes in Australian-Canadian OA Index (AUSCAN, German version) dimensions for pain and hand disability at week 52. The co-primary radiographic endpoint is the radiographic progression from baseline to week 52. A multiple endpoint test and analysis of covariance will be used to compare changes between groups. All analyses will be conducted on an intention-to-treat basis.

**Discussion:**

The OA TREAT trial will examine the clinical and radiological efficacy and safety of HCQ as a treatment option for inflammatory and erosive OA over 12 months. OA TREAT focuses on erosive hand OA in contrast to other current studies on symptomatic hand OA, for example, HERO [Trials 14:64, 2013].

**Trial registration:**

ISRCTN46445413, date of registration: 05-10-2011.

## Background

Osteoarthritis (OA) is a heterogeneous group of conditions with defective integrity of articular cartilage and changes in the underlying bone. The pathogenesis is multifactorial and involves a complex interplay of genetic, metabolic, biochemical, and biomechanical factors with variable components of inflammation. Subsets of patients with OA develop an inflammatory and erosive form of the disease. As one of the most prevalent musculoskeletal diseases, the condition leads to pain in and around the affected joints and to swelling, stiffness, deformity, and gradual loss of function [1, 2]. This disease not only affects older people, but also the younger working population. The pathologic mechanisms and its triggers are still not known. Treatment options are limited to symptomatic therapy and (rarely) surgical intervention. Compared to the research results of hip OA and knee OA, there are very few basic research activities in the field of hand OA. However, over the past 15 years, there has been an increase in the number of clinical trials in OA including hand OA. Nevertheless, effective treatments for OA are limited, since many treatments have only small symptom-relieving effects. There are few therapeutic studies in patients with hand OA investigating oral non-steroidal anti-inflammatory drugs (NSAIDs), topical NSAIDs, and other topical drugs. For the symptomatic slow-acting drugs in OA (SYSADOA) and disease modifying drugs in OA (DMOADS), very few studies can be identified, and unfortunately, many publications are available only as abstracts. Most of these randomized controlled trials (RCTs) used a broad range of outcome measures, some of them poorly standardized. Differences in disease definition between studies make it hard to generalize the results for clinical practice. Many studies were underpowered or planned as pilot studies [3, 4]. HCQ (originally an antimalarial drug) is a disease modifying drug (DMARD) used for inflammatory erosive OA in clinical practice. HCQ has been used since the 1950s for the treatment of various rheumatic and dermatologic diseases. Current research has further enhanced our understanding of the pharmacologic mechanisms of these drugs, involving inhibition of endosomal toll-like receptor (TLR) signaling, which inhibits B cell and dendritic cell activation [5]. With this understanding, the use of these medications in rheumatology is broadening.

In OA and hand OA, symptomatic therapy with NSAIDs or coxibs is the most frequently administered treatment. Regarding tumor necrosis factor (TNF)-α-antagonists, one pilot study with adalimumab (ADA) in patients with erosive/inflammatory OA (EOA) of the hands demonstrated that ADA was well tolerated; however, treatment with ADA for three months did not significantly improve signs and symptoms of EOA, and most patients did not achieve an ACR20 [6]. Three studies of HCQ in EOA, including one abstract and one letter, are available. HCQ at a dose of 200 to 400 mg daily was effective in seven patients with evidence of EOA on X-ray. This study was an open retrospective study [7]. The result of an open retrospective study of eight patients with EOA of proximal interphalangeal (PIP) and distal interphalangeal (DIP) joints and a dosage of 400 mg daily demonstrated that in six patients an improvement of synovitis, morning stiffness, and global assessment was seen [8]. One prospective, randomized, double-blind, and placebo-controlled trial in 15 patients with EOA of PIP and DIP joints and radiological changes consistent with OA showed that HCQ was more effective than placebo on clinical (Ritchie-Index) and biological assessments (ESR, IL_2receptor level) [9]. To summarize, the review of published data on hand OA raises more questions than it answers with regard to the evaluation of therapeutic agents in hand OA. Moreover, the few RCTs that have been performed were in small patient populations and lack standardized outcome assessments [4]. A task force of the Osteoarthritis Research Society International (OARSI) recently published guidelines and recommendations for clinical trials in hand OA [10]. Despite initial evidence for good efficacy of HCQ, there has not been a randomized, double-blind, and placebo-controlled trial in a larger patient group. In the European League Against Rheumatism (EULAR) evidence-based recommendations for the management of hand OA, HCQ was not included as therapeutic option because of the current lack of randomized clinical trials [11].

The evidence of HCQ in hand OA has never been investigated in controlled randomized studies, although there are clinical indications for good effectiveness in daily rheumatologic practice. Until now, HCQ is only used for hand OA in daily practice once all other available therapies have failed. HCQ treatment of patients with other rheumatic diseases shows that the drug is very well tolerated if management for the prevention of possible side effects (e.g. retinopathy) is carried out adequately. *In vitro* studies have shown that HCQ decreases the production of TNF-α, IL-6 and IFN-α by mitogen-stimulated peripheral blood lymphocytes [12]. A dose-dependent inhibition of TNF-α, IL-1β, and IL-6 by endotoxin-stimulated whole blood was also noted [13]. Monotherapy of systemic lupus erythematosus (SLE) patients with chloroquine results in a decrease in serum levels of IL-6, IL-18, and TNF-α [14]. It has been suggested that inhibition of TNF-α production by antimalarial drugs, which mainly affect monocytes, may be independent of the lysomotropic action of the drugs and related to nuclear effects [15]. HCQ acts as prostaglandin antagonist by inhibition of phospholipase A2 [16]. Rheumatoid arthritis (RA) and inflammatory OA synovial tissue have a similar pro-inflammatory and anti-inflammatory cytokine profile. OA cartilage shows lower production of proteoglycans, type II collagen, and IL-1β [17]. Moreover, HCQ potentiates Fas-mediated apoptosis of synoviocytes [18]. This background and the knowledge of the effectiveness in RA patients raise the question of whether this drug may also be effective in hand OA. When compared with other immunomodulatory agents, antimalarial drugs have a favorable safety profile. Our understanding of the toxicities and modes of action of these drugs may suggest new applications and modified treatment regimes in hand OA where there is huge unmet clinical need. On the other hand, more studies are needed to further explore the relationship between self-reported and radiographic outcomes and the relationship with other domains such as biomarkers and other imaging modalities [10, 19–21].

The aim of OA TREAT is to investigate the efficacy of HCQ by clinical and radiological outcomes compared to placebo in patients with severe and refractory inflammatory hand OA. The co-primary hypotheses are that patients receiving HCQ have a lower Australian-Canadian OA Index (AUSCAN) score in the dimensions for pain and hand disability at week 52 and that they have a lower rate of radiographic progression from baseline to week 52 compared to patients receiving placebo.

## Methods/Design

### Trial design

The trial is based on a call of investigator initiated trial funding 2009 by the German Ministry of Education and Research (Bundesministerium für Bildung und Forschung [BMBF]) and is carried out with German rheumatologic and statistical stakeholders with experience of treating hand OA. OA TREAT is a multicenter, double-blind, placebo-controlled phase III trial with a parallel group design.

### Study setting

Recruitment aims are based on the design of the study as a national multicenter study and on the established cooperation with primary care physicians within the Regional Collaborative Arthritis Centers (Department of Rheumatology and Clinical Immunology, Charité - Universitätsmedizin Berlin, German Competence Network Rheuma, HIT HARD Trial Network). All selected centers are very experienced in trial performance and approved by the local ethic committees (EC) in their quality management as a clinical trial center. Our partners are listed on our website for study (http://insider.charite.de/projekte/aktuelle_projekte/oa_treat/study_centers/).

### Participants and recruitment

Patients with hand OA according to the classification criteria of the American College of Rheumatology (ACR) with recent X-ray of the hands [22], dating from less than six months and showing radiological signs of digital erosive OA as defined by grades 2 or higher, per the Kellgren and Lawrence scale in one or more joints [23].

Participants must meet the inclusion and exclusion criteria in order to participate. These will be assessed at the screening visit. The key inclusion and exclusion criteria are listed in Table 1.

**Table 1:** **Key selected inclusion and exclusion trial criteria for patients**

Key inclusion criteria	Key exclusion criteria
1. Men and women from 40 to 80 years of age.	1. Patients who are currently treated with hydroxychloroquine (HCQ) or have received HCQ in the past due to OA of the hands.
2. Presence of clinical hand osteoarthritis (OA) according to American College of Rheumatology (ACR) criteria.	2. Patients who have not tolerated HCQ (for example, skin disease or malaria prophylaxis) or patients for whom HCQ was discontinued due to an eye disease.
3. Conforming to the ACR criteria for hand OA supported by X-ray of both hands, dating less than 6 months previous, X-ray of the hands showing radiological signs of digital erosive OA in one or more joints. This criterion is checked by a central assessment.	3. Existence of a pain syndrome of the upper limbs, which would interfere with the monitoring of pain.
4. Symptoms of digital inflammatory OA (pressure pain of the joint and/or florid joint swelling and/or redness and/or warmth) with more than three fingers’ joints for more than 3 months (at least every other day) despite taking analgesics and non-steroidal anti-inflammatory drugs (NSAIDs).	4. Patients suffering or having suffered from secondary OA after one of the following disease (for example, infectious arthritis, acromegaly, ochronosis, hemochromatosis, gout, *etcetera*) or inflammatory joint diseases.
5. Pain above 4 as evaluated by the Australian-Canadian OA Index (AUSCAN)-numeric rating score (NRS) (0-10).	5. Planned surgery.
6. Function as co-primary clinical outcome with ≥26 using the AUSCAN.	6. Local injection of finger or hand joints with glucocorticoids or other medications in the previous 3 months.
7. The ability to understand the trial information for patients (Arzneimittelgesetz, German Medicinal Products Act (AMG) §40(1) and 3b).	7. Current intake of oral, intra-articular (i.a.) or systematic glucocorticoids (intravenous (i.v.), intramuscular (i.m.)).
8. The ability to sign the written informed consent form including the data protection form (according AMG §40(1) and 3b).	8. Presence of retinopathy.
	9. Known hypersensitivity to HCQ or to one of the drugs in this study protocol.
	10. Treatment with digoxin
	11. Any unstable medical condition or other serious clinical situations that expose the patient to risk in the opinion of the local investigator.
	12. Current participation in another clinical trial or undergoing an experimental treatment.
	13. Patients who are underage or incapable of understanding the aim, importance and consequences of the study or of giving legal informed consent (according to AMG §40(4) and 42(2) and (3)).
	14. Prisoners and persons who are institutionalized due to AMG §40(1), no. 4
	15. Pregnant and breastfeeding women

A total of 510 patients with erosive and inflammatory hand OA will be recruited and randomly allocated to either the treatment or placebo group. Recruitment methods will include advertisements through the local media and community groups, invitations to previous study participants who have given their consent to be contacted regarding future research projects and liaisons with general practitioners, rheumatologists, orthopedists, and German patients associations (for example, Rheumaliga).

### Intervention

**Figure 1:**
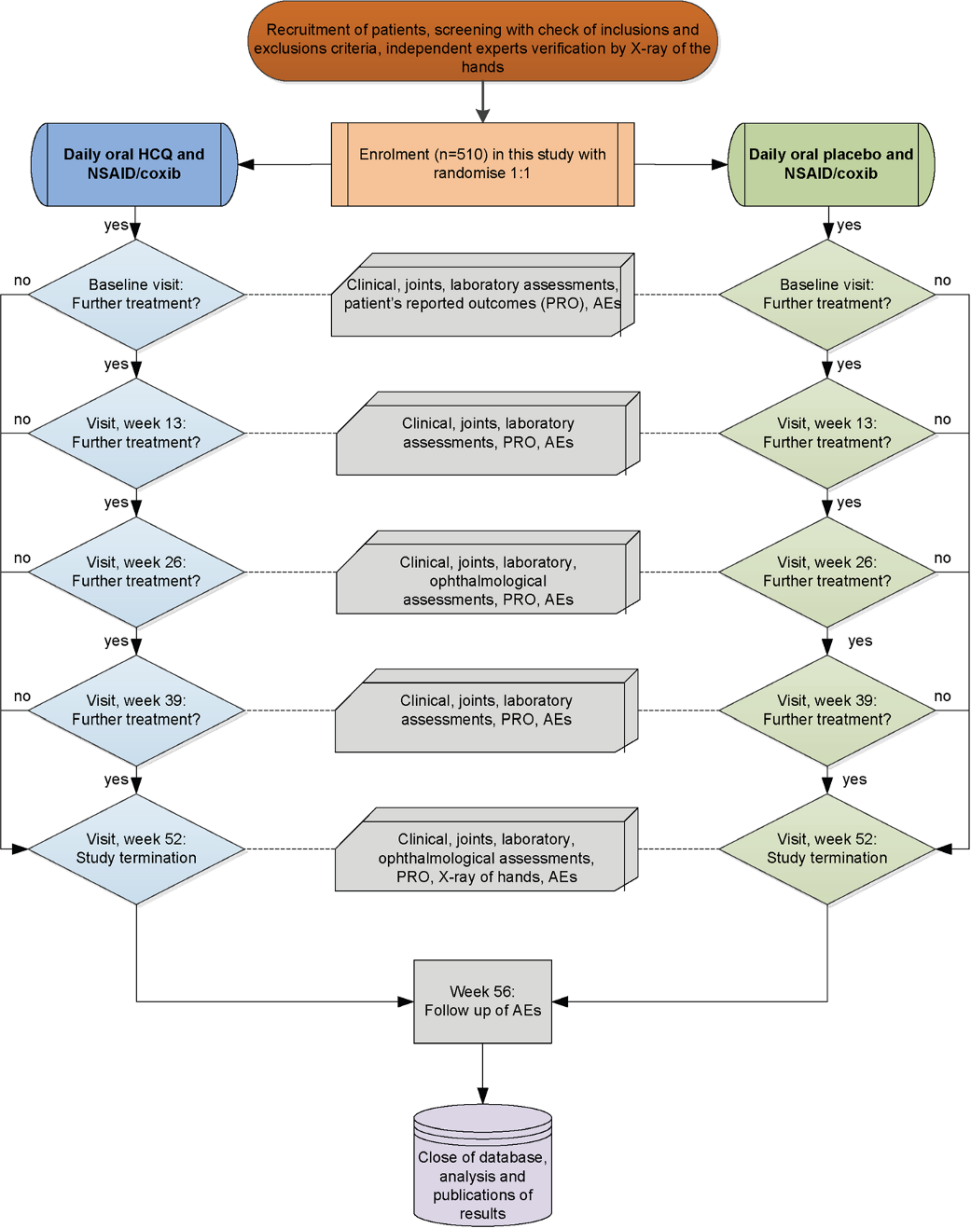
**Trial overview.**

Both groups receive standard therapy (NSAID, coxibs) for OA treatment, taken steadily two weeks before enrollment and continued afterwards (Figure 1). If disease activity increases, the dose of NSAID/coxibs can be increased according to the drug recommendation.Group I receives capsules with 200 to 400 mg HCQ as an oral application from day 1 up to the end of week 52. Group II receives capsules as an oral placebo application from day 1 up to the end of week 52. In both groups, a radiological examination is performed at week 52 (Figure 1). Rescue treatment with acetaminophen (maximum of 4 × 500 mg/d) is possible. Ongoing physiotherapy or occupational therapy before screening can be continued unchanged, but must not be commenced after enrollment.

The time schedule of enrollment, interventions, trial assessments, and visits for participants is described in Table 2.

**Table 2:** **Trial timeline, interventions, assessments and visits of OA TREAT**

Primary outcome measures:	
*Clinical endpoint*	Australian-Canadian Osteoarthritis (OA) Index (AUSCAN, German version) dimensions for pain and hand disability as co-primary clinical outcomes at week 52.
*Radiographic co-primary endpoint:*	Radiographic progression from baseline to week 52.
Key secondary endpoints:	
*Clinical outcomes:*	•Assessment and comparison of the inflammatory status using the following parameters: joint pain and joint swelling, night pain, morning stiffness, local erythema/redness, c-reactive protein (CRP) and erythrocyte sedimentation rate (ESR) levels from baseline to week 26,52.
	•Comparison of the increase or decrease of the consumption in the standard medication in the previous 7 days at each visit (non-steroidal anti-inflammatory drugs (NSAIDs), coxibs).
*Patients-reported outcomes*	•Patients global assessment of disease activity, patient’s assessment of stiffness, and physician’s global assessment of disease activity from baseline to week 26,52.
	•Comparison of pain, functioning, disability, quality of life, patient-acceptable symptoms and health with Health Assessment Questionnaire (HAQ), Short Form for the Changes of Quality of Life (SF-36), Short Form for the Assessment and Quantification of Chronic Rheumatic Affections of the Hands (SF-SACRAH), Australian-Canadian Osteoarthritis Index (AUSCAN), *etcetera* from baseline to week 26, 52.
*Assessment of safety*:	
Safety and tolerability of hydroxychloroquine (HCQ) with reports on adverse events (AE) and serious adverse events (SAE) from screening to baseline and week 12, 26, 39, 52, and follow-up of the eyes will be performed by an ophthalmologist at baseline and every 6 months (week 26 and 52).	

### Outcomes

OA TREAT examines a number of clinical, radiographic and patient-reported outcomes. These are listed in Table 3.

**Table 3:** **Primary and secondary outcomes of OA TREAT**

	Enrollment -30 days	Allocation/baseline 0	Post-allocation				Close-out week 52	Follow-up +28 days
Time point			Day 0	Week 13	Week 26	Week 39		
**Enrolment: Eligibility screen**	X							
**Informed consent**	X							
**Key investigations**								
•Medical history								
•Medication history								
•Clinical, laboratory and immunological investigation								
•Pregnancy test								
•X-ray of both hands and assessment by two blinded reviewers	X							
•Ophthalmological examination								
•AUSCAN Index								
•Patient-reported outcomes (HQA, SF-36, SF-SACRAH)								
**Allocation**		X						
**Interventions:**								
• Hydroxychloroquine			X*	X*	X*	X*	X*	
• Placebo			X*	X*	X*	X*	X*	
**Assessment:**								
**Baseline variables:**								
•Medical history								
Medication history								
•Clinical, laboratory and immunological investigation		X						
•AUSCAN-Index								
•Patients-reported outcomes (HAQ, SF-36, SF-SACRAH)								
•Safety (AE, SAE)								
•Accompanying research*								
**Outcome variables**								
•AUSCAN			X	X	X	X	X	
•X-ray of both hands							X	
Medication history								
•Laboratory and immunological investigation			X	X	X	X	X	
•Patient-reported outcomes								
•Accompanying research								
•Safety (AE, SAE)			X	X	X	X	X	X

X*: The therapy is administered continuously from day 1 to week 52.

AUSCAN: Australian-Canadian OA Index (German version) for pain and hand disability. The patient-centered self-administered AUSCAN Index is a valid, responsive and feasible tri-dimensional (pain, stiffness, and function) index developed specially for hand osteoarthritis studies [24–26].

HAQ-DI: Health Assessment Disability Index. The HAQ-DI assesses the degree of difficulty experienced in eight categories of daily living activity using 20 questions. This questionnaire has been validated for rheumatoid arthritis (RA); there is no experience in OA hand trials.

SF-SACRAH: Short Form Score for the Assessment and Quantification of Chronic Rheumatic Affections of the Hands. The SACRAH includes visual analog scales covering the extent of hand function, stiffness and level of pain from 0 to 100 [27]. The smallest number of questions of the modified score for the assessment and quantification of chronic rheumatic affections of the hands (M-SACRAH) providing reasonable reliability was identified by factor analysis and calculating Cronbach’s alpha, subsequently resulting in a five-item scale, the short form-SACRAH (SF-SACRAH) [23, 27].

SF-36: Short form for the Changes of Quality of Life. The SF-36 v.2 is a 36-item generic health status measure. It measures eight general concepts: physical functioning, physical role, bodily pain, general health, vitality social functioning, emotional role and mental health. These concepts can also be summarizes as physical (PCS) and mental component (MCS) scores. This questionnaire should be completed by the subject prior to any procedures being performed at the visit, if possible. To date, there is no experience in OA hand trials. Examples of health-related quality instruments include the SF-36 [28].

AE: Adverse events.

SAE: Severe adverse events.

*: Accompanying research of biomarkers in hand OA, dental and periodontal disease, health economic cost analysis.

### Methodological aspects

#### Sample size

Our sample size considerations are based on the EULAR recommendations for the planning of RCTs in patients with hand OA [13]. We considered differences in change scores in the AUSCAN scales pain and hand function, which were observed in RCTs comparing NSAIDs (lumiracoxib or ibuprofen) with placebo as clinically relevant differences that also should be detected with a sufficient power in this RCT. For these reasons, we aim to detect an effect size (ES) of 0.4 for pain (SD: 4.3) and of 0.25 for function (SD: 7.3) [13, 29]. An ES of 0.25 is also considered to be relevant for changes in the radiographic scores. Based on these assumptions, a sample size of n=255 per group is sufficient to achieve a power of 95% for the multiple endpoint test (first primary hypothesis) and an 80% power for the second primary endpoint (radiographic progression). The sample size is also sufficient to achieve an at least 80% power when comparing the two AUSCAN subscales pain and hand function separately.

#### Randomization, treatment assignment and allocation concealment

Randomization will be undertaken during normal working hours (Monday to Friday from 9:00 to 17:00) by the German Rheumatologic Research Center (Deutsches Rheumatologisches Forschungszentrum, DRFZ) of participating centers upon receipt of a randomization request form and after blinded review of X-rays of both hands.

Identical HCQ and placebo capsules will be produced to ensure allocation concealment. Upon production, study medication will be packed into numbered bottles according to a randomization schedule. This will be prepared by the DRFZ using a computerized random number generator, thereby guaranteeing full allocation concealment. Trial medication will be issued in numerical order by the pharmacy of the Charité - Universitätsmedizin Berlin. Participants in each site will be randomly assigned to the intervention arm or the placebo arm in a ratio of 1:1 and the randomization will be double-blind.

#### Blinding

As this is a double-blind study, all parties involved including the investigator, study coordinator, and subject will remain blinded to each subject’s treatment throughout the course of the study. The blind will be maintained at all times until all data has been collected and the study database locked. Participants will be randomized centrally by computer generated random numbers stratified by center. Patients and investigators are blind to the treatment allocation. A sealed envelope containing the blinded information will be provided to the investigator and retained by him/her in a locked or secured area. This envelope may be opened in the case of emergency. Unopened envelopes will be returned to the sponsor at the end of the study. Use of NSAIDs and rescue medication (acetaminophen) will be assessed carefully throughout the trial. The possible influence of these drugs on the outcome will be discussed. Radiographs will be read in a concealed time order by two readers blinded to treatment assignment.

#### Statistical methods

All patients who received at least one dose of the study medication will be included in the statistical analysis of the efficacy and safety of HCQ (modified intention-to-treat (mITT) population). To control the familywise error rate of 5%, a closed test procedure was applied [30]. In the first step, the first primary hypothesis is tested by means of the multiple endpoint tests according to Läuter and O’Brian [31]. Using this test the outcome in the AUSCAN scales pain and hand function at week 52 is compared between the treatment groups. The multiple endpoint test compares a standardized sum of both outcome measures (AUSCAN pain and function). Since we can assume that the outcome in pain and hand function are correlated, this test is more powerful than a Bonferroni-type test procedure or Hottelling’s T^2^ test [30, 32]. In the case of a significant difference of the first primary hypothesis, the second primary hypothesis (radiographic outcome at week 52) will be tested by means of an analysis of covariance (ANCOVA) based on ranks (nonparametric ANCOVA). Parametric ANCOVA with baseline status as co-variable will be applied to compare secondary outcomes (pain, function, quality of life parameters (SF-36)). Changes in the co-use of NSAIDs and/or analgesics will be compared between both groups. To estimate whether any imbalance in the use might have influenced the outcome in pain and function the suggestions by Dougados *et al*. for comparing different drugs and different dosages will be considered [33]. In the unlikely case that in the verum arm the dosages are more frequently increased by 50% or more (including an increase from 0 mg to half of the full dosage), this finding will be taken into account in the interpretation of the results.

#### Data collection and data management

All subject data obtained during the study will be recorded using pseudonyms. Every patient will be clearly identified by a registration number and a pseudonym, which will be assigned during the registration process. The investigator keeps a confidential patient list, in which the patient number and pseudonym are connected to the complete patient’s name. Access to this list is only provided to the local study personal and the study monitor. The original source files and medical records directly related to the study can be accessed by study monitors, auditors or inspectors from regulatory authorities. All data collection of pseudonomized data will be done on standardized case report forms (CRFs), which will be completed by site staff, verified by the principal investigator, and returned to the clinical trials unit for double data entry. All data will be recorded electronically at the sponsor’s site by independent data managers. The sponsor will maintain a list of personnel authorized to enter data into the database. Validation of correct data entry will be ensured by the named data managers to check for discrepancies and to ensure consistency of the data. Validated data will be entered into an electronic trial database. Any addition, change or correction to the entered data must be approved by the investigator and will be recorded. Records of correction will be kept together with the case report form. Regular backups of the electronic data will be performed. When all data have been recorded and validated after termination of the study the database will be closed. This process will be recorded.

The sponsor maintains a list of personnel authorized to access the data.

#### Monitoring and auditing

The monitor has the responsibility to familiarize the investigator(s) and the entire center staff involved in the study with all study procedures including the administration of the study drug. The sponsor must provide a trained monitor to assist the investigator(s) in conducting the clinical study. The monitor must visit the clinical study center on a regular basis and at least once before the first subject has been enrolled, once during the course of the study, and finally at study completion. The monitor has the responsibility of reviewing the ongoing study with the investigator(s) to verify adherence to the protocol and to deal with any problems that arise. At all times the sponsor must maintain the confidentiality of the study documents. It is the responsibility of the study monitor to verify the study documents against the subject’s original medical records. The investigator (or his/her deputy) agrees to cooperate with the monitor to ensure that any problems detected in the course of these monitoring visits are resolved. The harmonized SOP ‘Monitoring’ of the trial master file (TMF) (http://www.tmf-ev.de) is utilized for monitoring. The trial monitoring requires a high degree of professional expertise of the monitor. We have a variety of very specific outcome criteria, which are new to the involved trial sites. The focus of monitoring will be on developing, checking and adjusting the trial procedures, and on providing training, mentoring and support to study staff in response to the issues identified. In addition to the on-site monitoring, the following activities are planned: central monitoring of fax data and data management, training of and information on trial staff, and specific support of trial sites (information and download website) and investigators (investigator meetings). Independent audits will also take place in the course of study. The monitoring will be performed for 33% of source data verification, 100% of inclusion and exclusion criteria and 100% of primary endpoint criteria. Pre-study visits prior to the start of the study were carried out for 6 months in advance. A screening visit and six monitor visits per trial site are planned.

### Safety aspects

#### Data Monitoring and Safety Board

The Data Monitoring and Safety Board (DMSB) will be independent of both the investigators and the sponsor. The DSMB will review the blinded safety data, including adverse events reports. The DSMB will be empowered to request the unblinding of the subject’s treatment assignment or to recommend to the study team that this subject should be withdrawn from the study - regardless of the treatment assignment - if an emerging safety signal is suggested. The constitution, governing principles, and mandate of the DSMB are explained in the DSMB charter. The main tasks of the DSMB are to review relevant information about the trial; to ensure adherence to protocol; to advise whether to continue, modify or stop a trial; and to provide the funding organizations with information and advice.

#### Definition of unexpected adverse reactions

An unexpected adverse reaction (UAR) is one that is not listed in the Summary of Product Characteristics or in the Investigator Brochure (SmPC or IB). A UAR includes any event that may be symptomatically and pathophysiologically related to an event listed in the labelling, but differs from the labelled event because of greater severity or specificity.

#### Definition of suspected unexpected serious adverse reactions

Suspected unexpected serious adverse reactions (SUSARs) are serious events that are not listed in the IB or SmPC and that the investigator identifies as related to the investigational product or procedure.

A SUSAR is defined by the following criteria:

-  Description and severity of the SAE is not listed in the product labeling (IB or SmPC; GCP-V § 3(9)) and is
-  serious:
  1. Life-threatening condition or death.
  2. Initial inpatient hospitalization or prolongation of hospitalization required.
  3. Significant or persistent or disability or incapacity.
  4. Congenital anomaly/birth defect (including that occurring in a fetus).
  5. AE (GCP-V § 3(7)).

#### Assessment of severity

For both serious and non-serious AEs, the investigator must determine both the intensity of the event and the relationship of the event to study drug administration. Intensity for each adverse event will be determined by using the Common Terminology Criteria for Adverse Events (CTCAE, version 4.03) of the National Cancer Institute of the USA as a guideline, wherever possible. In those cases where the CTCAE criteria do not apply, the intensity of an adverse event will be defined according to the following criteria:

- Grade 1 - Mild: causing no limitations of usual activities
- Grade 2 - Moderate: causing some limitations of usual activities
- Grade 3 - Severe: causing inability to carry out usual activities.
- Grade 4 - Life-threatening: potentially life-threatening or disabling; urgent medical intervention required

In addition, for each AE, the investigator must assess whether the criteria for an SAE are fulfilled.

#### Documentation of adverse events and severe adverse events

Any severe adverse event (SAE) and any adverse event (AE) must be documented, whether it may or may not be considered related to the study drug or the study procedures. The documentation includes the nature of the event, the time point of occurrence, the duration, the severity and the causal relationship of the event.

When recording an AE, the investigator should use the overall diagnosis or syndrome using standard medical terminology, rather than recording individual symptoms, signs or changes in laboratory parameters. Laboratory parameters out of normal range must be assessed by the investigator with regard to the clinical relevance, and in case of clinical relevance, must also be reported as an AE. Any SAE and SUSAR, whether deemed treatment-related or not, must be reported by telephone and fax to the sponsor within 24 hours after the investigator or coordinator has become aware of its occurrence. The sponsor will ensure that all relevant information about SAEs and SUSARs that are fatal or life-threatening is recorded and reported as soon as possible to the national authorities (Federal Institute for Drugs and Medical Devices; Bundesinstitut für Arzneimittel und Medizinprodukte; BfArM) and to the responsible EC.

All other SAEs and SUSARs will be reported to the BfArM and to the EC as soon as possible but within a maximum of 15 days of first knowledge by the sponsor. The sponsor will also inform all investigators.

#### Annual safety reports

Once a year throughout the clinical trial, the sponsor will provide the national authorities BfArM and the EC with a listing of all SAEs and SUSARs that have occurred over this period and a report of the subjects’ safety.

#### Safety profile of hydroxychloroquine

The treatment is generally considered as low risk, and most side effects are not dangerous. The drug has been used successfully for several years in rheumatology and for malaria prophylaxis.

Patients report frequent side effects such as loss of appetite, flatulence, vomiting, diarrhea, headache, nausea, weight loss or unusual tiredness. These symptoms occur mainly during the first weeks of treatment.

Uncommon side effects include dizziness, blurred vision, emotional lability, corneal clouding, hearing impairment, hearing loss, drowsiness, numbness, sleep disturbances, dizziness, tinnitus, confusion, agitation, headache, paresthesia, decreased muscle strength, weakening of the tendon reflexes, changes in sensory perception, ECG changes, conduction disorders, heart disease, and nerve-muscle diseases.

Among the rare side effects are retina problems with visual field defects, impaired color vision, blurred vision, vision loss, changes in skin color, mucous membrane discoloration, bleached hair, gray hair, alopecia, pruritus, photosensitivity, liver problems or failure, beginning of psoriasis, the beginning of porphyria or exacerbation of myasthenia gravis. The very rare side effects include skin rashes, seizures, and methemoglobinemia.

#### Contraindications

Eye diseases with visual field defects, retinopathy, myasthenia gravis, bone marrow suppression, glucose-6-Ph-dehydrogenase deficiency, known allergy to the substance, and breastfeeding are contraindicated. Only under strict indications should HCQ be used when there are concerns about hepatic and renal function, porphyria, psoriasis, seizure disorders, or with concurrent use of monoamine oxidases (MAO) inhibitors in pregnancy. The following measures of therapeutic safety monitoring are provided in this trial:

1. Regular collection and reporting of all adverse events.
2. Medical control of the eyes at baseline and every 6 months.
3. Immediate notification of serious adverse event (SAE), SUSAR, suspected expected serious adverse reaction (SESAR), *etcetera* after becoming aware of the sponsor and follow-up reports within 7 days.
4. Regular meetings of the DSMB and decisions regarding the continuation of the study based on the AE reports.
5. Immediate treatments stop after decision of the investigator.
6. Regular monitoring of laboratory parameters of therapeutic safety.

### Ethics and dissemination

#### Regulatory approval and protocol amendments

Full ethical approval was granted by the independent EC of Berlin (13/029, Landesamt für Gesundheit und Soziales Berlin, LAGeSo) and by the BfArM. Full approval also was given by the Medicines and Healthcare Products Regulatory Agency (EudraCT 2011-001689-16).

The study will be announced to the LAGeSo Berlin. The sponsor and all participating investigators will be designated by their full name and position.

All protocol modifications must be prepared by a representative of the sponsor and initially reviewed and approved by the Trial Core Team (Medical Monitor, Trial Project Manager, Data Safety and Biostatistician) and Monitoring Boards. All protocol modifications must be submitted to the appropriate Independent EC of Berlin. The sponsor informs the BfArM and BMBF about all protocol modifications.

Approval must be awaited before any changes can be implemented, except for changes necessary to eliminate an immediate hazard to trial patients, or when the change(s) involves only logistical or administrative aspects of the trial (for example, change in monitor(s), change of telephone number(s).

#### Insurance

All participating patients are covered for the occurrence of study-related health damage. According to the German Drug Law, the sponsor of the study covers patient insurance.

#### Informed consent

Before the start of the study, the patients will be educated in detail - both verbally and in writing - about the study and the associated risks. Patients will have a decision time of at least 24 hours. It is the responsibility of the investigator, or a person designated by the investigator (if acceptable by local regulations), to obtain written informed consent from each patient participating in this study after adequate explanation of the aim, methods, anticipated benefits, and potential hazards of the study. For patients not qualified or incapable of giving legal consent, written consent must be obtained from the legally acceptable representative. In the case where both the patient and his/her legally acceptable representative are unable to read, an impartial witness should be present during the entire informed consent discussion. After the patient and representative have verbally consented to participation in the trial, the witness’ signature on the form will attest that the information in the consent form was accurately explained and understood. The investigator or designee must also explain that the patients are completely free to refuse to enter the study or to withdraw from it at any time and for any reason. The case report form (CRF) for this study contains a section for documenting informed consent, and this must be completed appropriately. If new safety information results in significant changes in the risk/benefit assessment, the consent form should be reviewed and updated if necessary. All patients (including those already being treated) should be informed of the new information, given a copy of the revised form and give their consent to continue in the study.

#### Study termination

Patients will be informed that they have the right to withdraw from the study at any time for any reason, without prejudice to their medical care. The investigator also has the right to withdraw patients from the study for any of the following reasons (but each patient with one application of HCQ and who is followed to X-ray investigation of the hands at week 52 is included in the trial analyses):

1. Intercurrent diseases.
2. Ophthalmologic changes associated with HCQ.
3. Occurrence of any grade 3 to 4 WHO toxicity or an unacceptable adverse event.
4. A treatment delay or interruption of *>*2 weeks because of toxicity.
5. Patient request.
6. Noncompliance.
7. General or specific changes in the patient’s condition unacceptable for further treatment in the judgment of the investigator.
8. Protocol violations.
9. Medical and ethical discretion of principal investigator.
10. Failure to return for follow-up.

The whole trial may be stopped for the following:

1. Administrative reasons.
2. Incidence of severe adverse events or fatal adverse events. Stopping rules are decided jointly by the data monitoring and safety board.

#### Procedure to avoid simultaneous inclusion in several studies

To avoid simultaneous inclusion in several studies, patients who had an experimental therapy or participated in clinical study with investigational medicinal products in the 3 months previous to the start of the treatment are excluded from this study. After a time interval of 3 months, inclusion of these patients is possible.

#### Confidentiality

Before the trial started, all local trial investigators gave written statements about conflicts of private, economical or financial interests with regard to the above-mentioned clinical trial and the investigational drugs that will be used.

#### Declaration of interests

The investigator will ensure that this study is conducted in full conformance with the principles of the ‘Declaration of Helsinki’ or with the laws of the country in which the research is conducted, whichever affords greater protection to the individual (in Germany for example, AMG/16.Novelle 2013). The study must fully adhere to the principles outlined in ‘Guideline for Good Clinical Practice’ ICH Tripartite Guideline (January 1997) or to local law if it affords greater protection to the patient.

#### Strategies for dissemination of results, dissemination policy

The results of the OA Treat study will consecutively improve the quality of rheumatologic treatment in Germany. The results will be analyzed and published independently of the pharmaceutical industry. Another important aspect is the implementation of the results of this study into the guidelines ‘Quality Assurance in Rheumatic Diseases’ and the ‘German Recommendations for Physicians - GC’ (http://www.dgrh.de), which have been developed by the working group of the German Society of Rheumatology (DGRh). The results of this study will be presented at scientific meetings and published in peer-reviewed scientific or medical journals. The study will be registered with the European Science Foundation as part of an effort to achieve European-wide registration of all randomized controlled clinical trials; it is possible that the results will spark international discussions in scientific circles to allow for the possibility of extrapolating the findings to other conditions. The cooperation with the Competence Network Rheumatology, the DRFZ and the various national trial centers enables us to use the results and experience in the field of other therapy options in the treatment of OA. Also, other medical disciplines, in which the disease is often treated, can benefit from these results. For this reason, the dissemination of the results in general medical journals and interdisciplinary conferences is planned.

## Discussion

Inflammatory and erosive OA of the hands is a chronic disease that severely impacts the quality of life not only in older people but also the younger working population. Treatment options are currently limited to symptomatic therapy and (rarely) surgical intervention, with NSAIDs or coxibs being the most frequently administered treatment. Unfortunately, their symptom-relieving effect is often too small, and adverse effects can be substantial. There is, therefore, huge unmet clinical need for effective and safe treatment options.

There are currently few therapeutic options to treat hand OA. Often NSAIDs are prescribed. In long-term use, however, these effective drugs have a very high risk of gastrointestinal bleeding and impairment of the renal function. Coxibs are alternatively available, but cardiovascular side effects have been reported for both NSAIDs and coxibs. Although clinical trials showed a trend for a good efficacy of HCQ in hand OA, the development of the drug was not pursued further. A major reason was the lack of interest from the pharmaceutical industry. In the treatment of RA and SLE, HCQ is a very effective drug and its side effect profile has been known for many years. Provided that all safety measures are carried out, this drug is very well tolerated and also safe in continuous treatment. Hand OA is not a life-threatening illness, but leads to a severely impaired quality of life due to pain. In addition to the definition of termination criteria, safety-related parameters are regularly investigated and questioned (for example, AE, SAE) during the study. Before the start of the study, the patients will be educated in detail about the associated risks. Patients will receive a decision time of at least 24 hours before giving written consent. Furthermore, the patient may withdraw his consent at any time during the study. The project will follow all regulations and legal requirements for data protection, AMG/GCP, and the Declaration of Helsinki.

The co-primary clinical endpoints in this study are the changes in AUSCAN dimensions for pain and hand disability at week 52. The AUSCAN (Australian/Canadian hand OA Index) is a patient-centered self-administered questionnaire developed specifically for hand OA studies [31]. It consists of three subscales and a total of 15 items: pain subscale (rest, gripping, lifting, turning, and squeezing), stiffness subscale (morning stiffness), and a physical function subscale (turning taps/faucets, turning a round doorknob or handle, doing up buttons, fastening jewelry, opening a new jar, carrying a full pot, peeling vegetables/fruits, picking up large heavy objects, and wringing out wash cloths) [31]. It has been validated in both OA and RA patients. Associations with grip strength and radiographic severity of hand OA have been demonstrated. Post-validation experience in phase III clinical trials has confirmed the responsiveness of the AUSCAN. Versions with 5-point Likert scales, 100-mm visual analog scales (VAS) and 11-point numerical rating scales (NRS) are available [31]. In this study, we chose the NRS version as it combines the simplicity of the Likert scale with the high responsiveness of the VAS. The most commonly used approach to analyze the questionnaire is by summation of the 15 components (total AUSCAN score), resulting in scores between 0 and 150 [31].

The following potential limitations of the study should be noted: generalizability of the results may be limited by specific inclusion and exclusion criteria, and results may not apply to the entire target population. However, selection criteria were kept as broad as possible to ensure maximal generalizability. For ethical reasons, changes in the NSAID dosages including new treatment starts or treatment terminations are allowed. Since an imbalance in the use of co-medications might have an influence on the outcome in the clinical parameters, the use of NSAID/analgesics is recorded at each study visit and will be analyzed as a secondary outcome. In case there is any imbalance, we anticipate an increased use of co-medication in the placebo group, which could weaken the differences between both treatment arms especially regarding the AUSCAN scales for pain and hand function. However, even if this increased use of NSAIDs/analgesics would reduce the assumed effect sizes for both scales by 25%, the power of the multiple endpoint test comparing the first primary outcome will be greater than 80%. In the unlikely case of an increased use of NSAIDs and/or analgesics in the verum arm, this observation will be considered in the interpretation of the findings to discuss the possibility of a false positive result.

HCQ has been used since the 1950s for the treatment of various rheumatic and dermatologic diseases. It is also used for inflammatory erosive OA in clinical practice, but there are no high-quality clinical trials to support its use in hand OA. Three pilot studies of HCQ in EOA are available, but their results are contradictory, population sizes are small, and there is a lack of standardized outcome measures. Despite a favorable safety profile and initial evidence for good efficacy of HCQ, there has not been a randomized, double-blind, and placebo-controlled trial in a larger patient group. HCQ was therefore not included as a therapeutic option in the EULAR evidence-based recommendations for the management of hand OA [11]. To close this gap, we are currently performing the trial OA TREAT to investigate the efficacy of HCQ by clinical and radiological outcomes compared to placebo in patients with severe and refractory inflammatory hand OA. In contrast to other current studies on symptomatic hand OA, for example, HERO [34], OA TREAT focuses on erosive hand OA, a more severe subset of hand OA with very limited therapy options.

## Trial status

Recruitment and follow-up of participants are in progress.

## Abbreviations

ACR: American College of Rheumatology
ACR20: ACR20 criteria
ADA: adalimumab
AE: adverse event
AMG: Arzneimittelgesetz, German Medicinal Products Act
ANCOVA: analysis of covariance
AUSCAN: Australian-Canadian OA Index
BfArM: Bundesinstitut für Arzneimittel und Medizinprodukte, Federal Institute for Drugs and Medical Devices
BMBF: Bundesministerium für Bildung und Forschung, German Ministry of Education and Research
CRF: case report form
CRP: c-reactive protein
CTCA: Common Terminology Criteria for Adverse Events
DGRh: Deutsche Gesellschaft für Rheumatologie e.V., German Society of Rheumatology
DIP: distal interphalangeal joints
DMARDs: disease-modifying anti-rheumatic drugs
DMOADS: disease-modifying drugs in osteoarthritis
DMSB: Data Safety and Monitoring Board
DRFZ: Deutsches Rheumaforschungszentrum, German Rheumatism Research Center
EC: Ethics Committee
ECG: electrocardiogram
EOA: erosive and inflammatory osteoarthritis
ES: effect size
ESR: erythrocyte sedimentation rate
EudraCT: European Clinical Trials Database
EULAR: European League Against Rheumatism
FKZ: Förderkennzeichen, reference number
GCP-V: Good Clinical Practice - Verordnung, German Regulation on Good Clinical Practice
HAQ: Health Assessment Questionnaire
HAQ-DI: Health Assessment Questionnaire, Disability Index
HCQ: hydroxychloroquine
HERO: Hydroxychloroquine Effectiveness in Reducing Symptoms of Hand Osteoarthritis
HIT HARD: High Induction Therapy with Anti-Rheumatic Drugs
i.a.: intra-articular
i.m.: intramuscular
i.v.: intravenous
IB: investigator brochure
ICH: International Conference on Harmonisation
IFN: interferon
IL: interleukin
LAGeSo: Landesamt für Gesundheit und Soziales, Independent Ethics Committee of Berlin
MAO: monoaminooxidase
mITT: modified intention-to-treat
NRS: numeric rating scale
NSAID: non-steroidal anti-inflammatory drugs
OA: osteoarthritis
OAR: Osteoarthritis Research Society International
PIP: proximal interphalangeal joints
PRO: patient-reported outcomes
RA: rheumatoid arthritis
RCT: randomized clinical trial
SAE: serious adverse event
SESAR: suspected expected serious adverse reaction
SF-36: Short Form for the Changes of Quality of Life
SF-SACRAH: Short Form for the Assessment and Quantification of Chronic rheumatic Affections of the Hands
SLE: systemic lupus erythematosus
SmPC: summary of product characteristics
SOP: standard operating procedure
SUSAR: suspected unexpected serious adverse reaction
SYSADOA: symptomatic slow-acting drugs in OA
TLR: toll-like receptor
TMF: trial master file
TNF: tumor necrosis factor
UAR: unexpected adverse reaction
USA: United States of America
WHO: World Health Organisation.
